# Corrigendum: Electrical Detection of Dengue Biomarker Using Egg Yolk Immunoglobulin as the Biological Recognition Element

**DOI:** 10.1038/srep46865

**Published:** 2017-06-30

**Authors:** Alessandra Figueiredo, Nirton C. S. Vieira, Juliana F. dos Santos, Bruno C. Janegitz, Sergio M. Aoki, Paulo P. Junior, Rodrigo L. Lovato, Maurício L. Nogueira, Valtencir Zucolotto, Francisco E. G. Guimarães

Scientific Reports 5: Article number: 786510.1038/srep07865; published online: 01 19 2015; updated: 06 30 2016

This Article contains an error in the legend of Figure 3.

“(a) Dynamic response of the proposed immunosensor upon the addition of 1 mg.mL^−1^ NS1 in the measuring cell.”

should read:

“(a) Dynamic response of the proposed immunosensor upon the addition of 1 μg.mL^−1^ NS1 in the measuring cell.”

